# Heavy metal – habitual licking of a lead tape as rare cause of chronic lead poisoning: a case report

**DOI:** 10.3389/fped.2026.1909793

**Published:** 2026-09-09

**Authors:** Franziska Carolin Lammert, Jens Rengelshausen, Jens Bertram, Thomas Kraus, Frederic De Beukelaer, Jens Nickel, Moritz Muschaweck

**Affiliations:** 1 Department of Pediatrics, RWTH Aachen University Hospital, Aachen, Germany; 2 Institute for Occupational, Social and Environmental Medicine, RWTH Aachen University, Aachen, Germany; 3 Clinic for Diagnostic and Interventional Neuroradiology, RWTH Aachen University Hospital, Aachen, Germany; 4 Health department Rhein-Erft-Kreis, Bergheim, Germany

**Keywords:** case report, chelation therapy, lead encephalopathy, lead poisoning, porphyria

## Abstract

**Background:**

Lead poisoning remains a major environmental health risk for children, as no safe blood lead concentration has been identified and even low-level exposure can cause neurocognitive, behavioral, cardiovascular and renal impairments. Although blood lead levels have declined substantially in high-income countries due to regulatory measures such as the elimination of leaded gasoline and stricter environmental controls, lead exposure continues to pose a significant global public health burden, particularly in low- and middle-income countries. Moreover, uncommon exposure pathways still occur in industrialized nations, making the diagnosis challenging and underscoring the importance of considering environmental exposures in pediatric practice.

**Case presentation:**

A 10-year-old boy presented with a two month history of colicky abdominal pain, constipation, postprandial vomiting, 7 kg weight loss, and general weakness. A diagnostic laparoscopy performed at an external hospital was unremarkable. He had undergone appendectomy two years earlier and had no prior health issues. During hospitalization he developed visual disturbances, headaches, and abdominal pain crises requiring opioid treatment. He exhibited psychotic behavior, but no evidence for somatization disorder was found.

**Results:**

Laboratory tests revealed mildly elevated aminotransferases, hypochromic microcytic anemia and basophilic stippling on the blood smear. Abdominal x-ray and MRI were unremarkable. Urinary porphyria analysis confirmed elevated porphobilinogen (PBG) and delta-aminolaevulinic-acid (ALA), suggestive of ALA-dehydratase-deficiency (Doss-porphyria). Cranial MRI showed lesions compatible with posterior reversible encephalopathy syndrome (PRES), consistent with previously described porphyria-induced PRES. As differential diagnosis, lead intoxication was considered, as it inhibits ALA-dehydratase and raises ALA more than PBG. Surprisingly, blood lead levels were markedly elevated (581 µg/L (≤22 µg/L)). Chelation therapy with dimercaptopropanesulfonic acid was initiated, leading in symptom resolution within 48 h. As no source of lead exposure was initially identified and family members showed no elevated levels, ongoing exposure was suspected. After seven weeks, the boy's grandmother discovered the source: a damaged lead tape used as curtain weight, which the boy had been licking for months while gaming. Following removal of this tape, blood lead levels gradually declined.

**Conclusion:**

Lead intoxication should remain a differential diagnosis despite its decreasing incidence. Its clinical presentation can mimic porphyria, and lead-induced encephalopathy may resemble PRES.

## Introduction

Lead poisoning remains one of the most important environmental toxic exposures affecting children worldwide (1). Due to increased gastrointestinal absorption and the vulnerability of the developing central nervous system, children are especially susceptible to adverse effects of lead exposure. Current evidence indicates that no safe blood lead concentration exists, and even low-level exposure has been associated with neurocognitive deficits, behavioral disturbances, impaired academic achievement, and long-term cardiovascular and renal morbidity (2–5). In high-income countries, including Germany and most parts of Europe, the epidemiology of pediatric lead exposure has changed substantially over the past decades. Following the elimination of leaded gasoline, stricter regulation of industrial emissions, restrictions on lead-containing consumer products, and improvements in environmental protection, blood lead concentrations have declined dramatically since the late twentieth century (1, 6, 7). Consequently, clinically manifest lead intoxication has become an uncommon diagnosis in routine pediatric practice. This epidemiological success, however, may paradoxically contribute to delayed recognition, as many clinicians have limited experience with the condition and its often-nonspecific presentation.

In contrast, lead exposure remains a major global public health problem. According to the UNICEF–Pure Earth report *The Toxic Truth*, approximately one-third of all children worldwide - up to 800 million individuals - have blood lead levels ≥5 µg/dL, with the overwhelming burden occurring in low- and middle-income countries (1, 3). Informal recycling of lead-acid batteries is considered one of the most important exposure sources globally, while additional contributors include mining and smelting activities, electronic waste recycling, lead-containing paints and pigments, contaminated spices, cosmetics, pottery, cookware, traditional medicines, and polluted soil and dust (2). Although environmental and occupational sources account for most cases worldwide, sporadic pediatric intoxications in Europe are increasingly linked to unusual or overlooked exposure pathways. Reported sources include ingestion of lead-containing foreign bodies, lead-based paint in older buildings, contaminated imported consumer products, fishing sinkers, ammunition, and lead pellets (2). Such atypical exposures may be difficult to identify, particularly in regions where lead poisoning has become rare and is therefore not routinely considered in the differential diagnosis.

We report the case of a 10-year-old boy who developed symptomatic lead intoxication after repeatedly sucking on lead-containing pellets. This case highlights the ongoing relevance of lead poisoning in high-income countries, emphasizes the importance of a detailed environmental exposure history, and illustrates the diagnostic challenges associated with an increasingly uncommon but potentially severe pediatric condition.

## Case description

A 10-year-old boy was transferred from a regional hospital suffering from severe colicky abdominal pain, constipation and bilious vomiting for over two months, accompanied by generalized weakness and weight loss of 7 kg over a six-week period. Oral food intake had become impossible. Over the previous two months, he had consulted several physicians multiple times; all of whom had attributed his symptoms to prolonged gastroenteritis. He even underwent a diagnostic laparoscopy, which revealed no pathologic findings. The boy was previously healthy, aside from an appendectomy performed two years earlier.

During pain crises, which occurred several times a day, he showed a presumably demonstrative psychotic behavior. The family no longer recognized their child, whose behavioral abnormalities and personality changes suggested an underlying psychiatric condition, such as somatization disorder. The patient had a high demand for pain medication, whereby the symptoms could not be fully reduced even with adequate dosages of NSAID, metamizole or piritramide. The pain crises were accompanied by arterial hypertension (maximum 155/115 mmHg). At this stage of the illness, a detailed assessment or examination of the boy was hardly possible due to the severe pain and the resulting psychological distress. We consulted child and adolescent psychiatrists, who were unable to diagnose an isolated somatization disorder.

### Diagnostic assessment

Our laboratory findings revealed slightly elevated transaminases (AST 62 U/L, ALT 76 U/L), bilirubin (direct 0.67 mg/dL; total 1.44 mg/dL), and *γ*GT (62 U/L), as well as hypochromic microcytic anemia with a minimal hemoglobin level of 6.4 g/dL (MCV 73.7 fL, MCH 24.9 pg), accompanied by elevated ferritin (431 ng/mL) and soluble transferrin receptor (5.59 mg/L) (Supplementary Table 1). Our patient required a blood transfusion on one occasion. We excluded acute infectious and autoimmune hepatitis, Wilson's disease, hemochromatosis, and coeliac disease. x-ray imaging, abdominal ultrasonography, and abdominal MRI were unremarkable.

**Figure 1:**
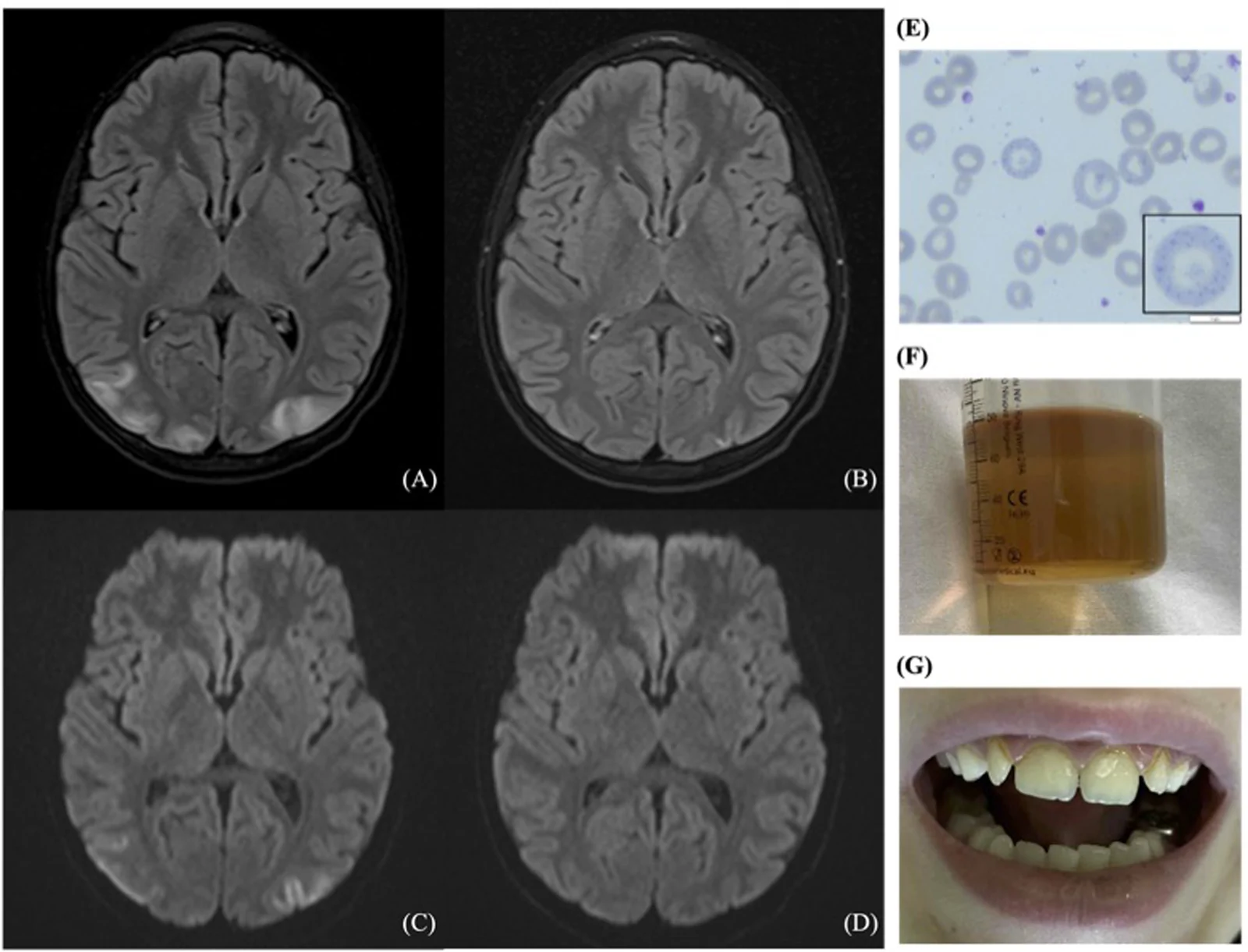
Pre- **(A,C)** and posttreatment **(B,D)** axial cMRI (1,5 T). *Top:* Fluid-attenuated inversion recovery (FLAIR) sequence. *Bottom:* diffusion weighted echo-planar imaging (EPI) sequence. Pretreatment images show cortical gray and subcortical white matter lesions in the occipital lobe with edema and sulcal effacement. Posttreatment images reveal near-complete resolution. Few focal cortical gray matter lesions in the left occipital lobe. **(E)** Blood smear showed basophilic stippling. **(F)** The patient's dark-colored urine after exposure to sunlight appears to be compatible with porphyria. **(G)** Discoloration of gingival margins.

Three days after admission, visual disturbances, such as blindness, blurred vision and headache occurred. The visual acuity could not be tested by the ophthalmologists due to the patient's lack of compliance. Subsequently, the EEG showed normal findings, while cMRI revealed prominent signs consistent with posterior reversible encephalopathy syndrome (PRES) or autoimmune encephalitis (Figure 1A). PRES is a rare but clinically significant syndrome that can present with headache, visual disturbances, acute arterial hypertension, and other neurological abnormalities such as impaired consciousness or seizures (8). It was therefore largely consistent with the boy's symptoms, except for the absence of seizures. Cerebrospinal fluid (CSF) analysis (3 cells/µL, protein 0.22 g/L, lactate 1.7 mmol/L, glucose 63 mg/dL) showed no evidence of blood-brain barrier dysfunction or oligoclonal bands. However, autoimmune encephalitis could not be definitively excluded. Therefore, we initiated immunoglobulin therapy (a total of 2 g/kg) over three days. In addition, treatment with acyclovir was carried out until the virus PCR from the cerebrospinal fluid was negative. Autoimmune antibodies could not be detected in the course of the disease.

As a rare differential diagnosis of chronic abdominal pain, constipation, and psychotic behavior, we considered porphyria. Moreover, porphyria has previously been described as a rare cause of PRES (9, 10). Therefore, we targeted biochemical screening for porphyria. Our screening revealed markedly elevated levels of *δ*-aminolaevulinic acid (ALA) at 49.62 mg/24 h (reference range: 0.25–6.4 mg/24 h) and slightly increased porphobilinogen (PBG) at 3.87 mg/24 h (reference range: 0.1–1.7 mg/24 h). Subsequently, we observed basophilic stippling of erythrocytes on blood smear (Figure 1E), dark-colored urine following sunlight exposure (Figure 1F), as well as discoloration of the gingival margins (Burtońs line) (Figure 1G), raising strong suspicion of porphyria. However, the patient´s biochemical profile, characterized by markedly elevated ALA with comparatively near-normal PBG levels (ALA to PBG ratio of 12.8:1) (Table 1) is consistent with ALA dehydratase deficiency porphyria (Doss-porphyria), an exceptional rare condition with only eight cases reported worldwide (11). Another cause of this constellation is an acute or chronic lead intoxication, which is also a rare condition but significantly more common and therefore more likely than ALA dehydratase deficiency porphyria. Lead inhibits the ALA dehydratase (ALAD), leading to the accumulation of heme synthesis intermediates – particularly ALA – and thereby mimicking clinical and biochemical features of porphyria (12, 13, 38) (Supplementary Figure 1).

**Table 1:** Laboratory values of porphyrin screening.

Parameter	Patient's value	Reference value
ALA	**124.3** µmol/24h	< 49.0 µmol/24h
ALA/creatinine	**46.89** mmol/mol	< 2.66 mmol/mol
porphobilinogen (PBG)	3.9 µmol/24h	< 7.5 µmol/24h
PBG/creatinine	**1.46** mmol/mol	< 1.01 mmol/mol
ALA/PBG-ratio	**32.22**	1.4–14.4
uroporphyrin	15.4 nmol/24h	< 32 nmol/24h
uroporphyrin/creatinine	**5.81** µmol/mol	< 4.33 µmol/mol
heptacarboxyporphyrin	5.6 nmol/24h	<10 nmol/24h
heptacarboxyporphyrin/creatinine	**2.11** µmol/mol	< 1.36 µmol/mol
pentacarboxyporphyrin	**9.3** nmol/24h	< 6 nmol/24h
pentacarboxyporphyrin/creatinine	**3.51** µmol/mol	< 0.81 µmol/mol
coproporphyrin	**1497 **nmol/24h	<153 nmol/24h
coproporphyrin/creatinine	**564.32** µmol/mol	< 20.68 µmol/mol
total porphyrin (urine)	**1529** nmol/24h	< 209 nmol/24h
total porphyrin/creatinine	**576.53** µmol/mol	< 28.28 µmol/mol
zinc protoporphyrin	**418.8** µmol/mol	< 40 µmol/mol
ALAD	**84** mmol/l/h	690-1282 mmol/l/h
ALAD activity after reactivation	**865** mmol/l/h	690-1282 mmol/l/h
reactivation factor	**10.3**	< 1.5
lead concentration (serum)	**894.4** µg/l	< 60 µg/l

Elevated/reduced parameters are marked in bold.

To rule out lead poisoning we measured lead concentrations on blood and urine samples. Strikingly, extremely elevated lead levels were found in the boy's blood (580.7 µg/L, reference value for 3-11-year-old boys: 22 µg/L) and urine (227.7 µg/L, no reference values available). In lead poisoning, both coproporphyrinogen oxidase and ferro chelatase may be inhibited, contributing to the development of anemia in affected individuals (14–16). These shifts in heme synthesis were also observed in our patient (Table 1). As a result of lead accumulation, disruptions in heme synthesis occurred due to enzyme inhibition and accumulation of intermediates – particularly ALA and coproporphyrin – which explained the patient's porphyria-like symptoms. Furthermore, lead poisoning accounted for the encephalopathy observed both clinically and on MRI, mimicking the presentation of PRES. Previously, lead encephalopathy similar to PRES was described in a 50-year-old patient (17).

### Therapeutic intervention and follow up

**Figure 2:**
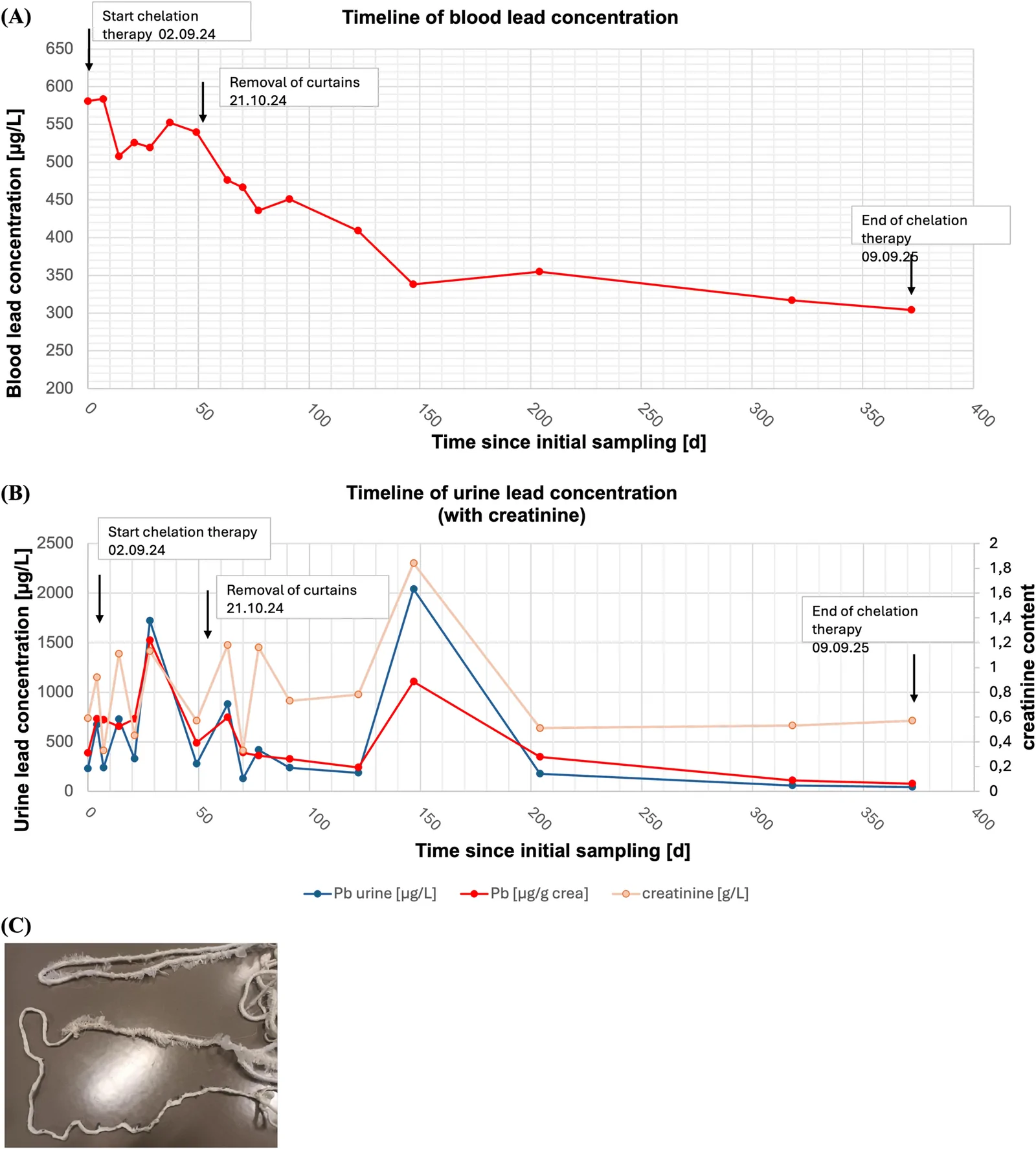
Lead concentration in blood **(A)** and urine **(B)** before, during and at the end of chelation treatment. **(C)** Curtain weights with lead-containing pearls, which served as the boy's oral lead source for months.

Intravenous chelation therapy with Dimaval® (dimercaptopropane sulfonic acid) was promptly initiated (initial dosage: 6 × 150 mg i.v.; long-term treatment: 3 × 200 mg p.o.). The treatment was well tolerated and resulted in a complete resolution of symptoms within 72 h. No recurrence of abdominal pain, vomiting, headache, or visual disturbances was observed thereafter. Lead excretion in the urine was checked during therapy. This showed significantly increased lead concentrations in urine (before treatment: 227.7 µg/L; during treatment: 988.4 µg/L), proofing the success of therapy. With intravenous therapy, the lead concentration in the blood dropped rapidly, reaching 508.0 µg/L after one week of treatment (Figure 2). Renal retention parameters remained within normal limits throughout the course of chelation therapy. Further analysis revealed significantly elevated zinc protoporphyrin levels (418.8 µmol/mol; reference value < 40.0 µmol/mol), indicating chronic lead intoxication (18). As expected, blood lead concentrations remained elevated over several weeks of therapy, since lead is deposited in the bone during chronic intoxication and is subsequently released back into the bloodstream through bone metabolism over months or even years (19). Follow-up cMRI scans after 1.5 and 4 weeks of therapy showed a marked reduction of encephalopathy, with residual parieto-occipital changes (Figure 1B).

## Discussion

Despite intensive efforts, the source of lead exposure could not be identified during hospitalization. Close relatives were tested for blood lead concentrations and showed no elevation, indicating that the boy appeared to be the only person affected. This unusual constellation prompted consideration of inherited ALAD deficiency, which could explain an increased sensitivity to lead accumulation (20). However, laboratory screening of the boy's heme synthesis parameters showed a possible reactivation of ALAD (Table 1), thereby making a genetic cause for the elevated lead levels almost impossible. After discharge, serum lead level remained very high (> 500 µg/L; Figure 2A), raising suspicion of ongoing lead exposure in the home environment. In order to identify the source, on-site inspections were conducted at the school and the family's residence by the local public health department. Water samples collected during these inspections showed only negligible levels of lead, excluding a common and well-recognized exposure pathway. After seven weeks of chelation therapy, the boy's grandmother discovered a damaged chain at home, used as a curtain weight, with lead-based pearl-like elements. The boy had been regularly licking and mouthing the chain for months while gaming – a habit he continued even during chelation therapy. The chain was identified as the ongoing oral source of lead exposure, explaining the persistently high serum lead level (Figure 2C). These curtain weights were analyzed and were found to contain a significant amount of lead (Supplementary Table 2). Following cessation of exposure, lead concentrations decreased after approximately seven weeks of chelation therapy.

This case underscores the importance of considering non-traditional consumer products as potential sources of lead exposure in children. Although regulations in many countries have substantially reduced lead in toys and children's products, lead-containing decorative items, paint, jewelry, cookware, traditional remedies, and imported consumer goods continue to represent underrecognized hazards. Such products often fall outside the scope of regulations specifically targeting children's articles, despite their potential accessibility to children within the household (1–3, 21–23). The delayed identification of the source in the present case further illustrates a common challenge in pediatric lead poisoning: environmental assessments frequently focus on traditional and established exposure routes such as contaminated water, paint, soil, or occupational take-home exposure, whereas behavioral habits involving repetitive mouthing of ordinary household objects may remain undisclosed unless specifically explored. Comparable reports by Toto and colleagues of unanticipated sources, including severe neurological presentations following the unrecognized ingestion of lead-containing metallic objects (e.g., metallic ring), reinforce this point (24). Detailed environmental history-taking, including direct questioning regarding pica-like behaviors, object chewing, and gaming-related habits, may therefore be critical in identifying hidden exposure sources.

However, the boy's blood lead levels remained elevated over time, even with ongoing chelation therapy. This slow reduction is consistent with the established toxicokinetic of lead. Following absorption, lead is transported predominantly within erythrocytes and distributed to soft tissues along pathways shared with calcium; a substantial proportion is subsequently incorporated into the skeleton (5, 25). Whereas the apparent half-life of lead in blood after a single exposure is approximately 30–35 days, its persistence in brain (on the order of two years) and bone is substantially longer and may extend over several years to decades (25–27). In chronically exposed children, the skeletal compartment may account for more than 70% of the total body lead burden, and therefore serves not only as a repository but also as an endogenous source of lead that can be remobilized into the circulation (5, 28). Importantly, the skeletal compartment is more labile in children than in adults because of ongoing bone growth and remodeling, potentially facilitating redistribution of stored lead into the circulation (5, 27). Thus, blood lead concentrations measured after cessation of external exposure may reflect both recent exposure and the ongoing release of previously accumulated lead from internal stores.

**Figure 3:**
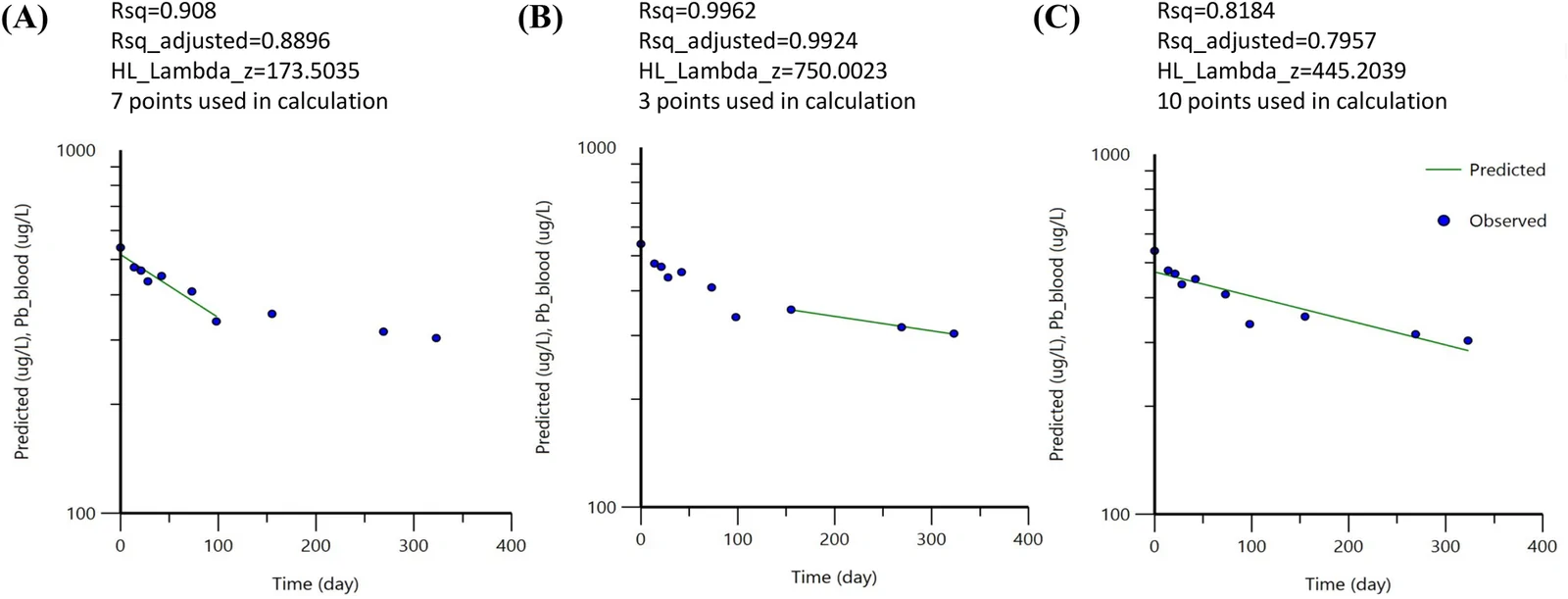
Non-compartmental analysis of blood lead elimination kinetics using log-linear concentration–time plots. Observed serum lead concentrations (blue dots) and corresponding log-linear regression lines (green) are displayed for the initial **(A)**, the terminal elimination phase **(B)**, and overall regression **(C)** Initial phase yielded an elimination half-life of 0.48 years (7 data points, R^2^ = 0.91), the terminal phase showed a markedly prolonged half-life of 2.05 years (3 data points, R^2^ = 0.996), overall regression resulted in an intermediate half-life of 1.22 years (10 data points, R^2^ = 0.82) and a substantially poorer fit.

Non-compartmental analysis of the plasma concentration–time data demonstrated a biphasic elimination pattern (Figure 3), with an initial blood elimination half-life of approximately 0.48 years followed by a markedly prolonged terminal blood elimination half-life of approximately 2.05 years. Both values exceed the half-life expected after a single exposure by an order of magnitude, which is itself informative: in a chronically exposed patient the measured decline in blood lead reflects the emptying of a large, slowly equilibrating skeletal reservoir rather than the clearance of a single absorbed dose. The substantially improved fit for the phase-specific regressions compared with the overall regression supports the presence of distinct elimination phases and renders calculation of a single overall elimination half-life biologically and methodologically inappropriate. The observed kinetics are consistent with the redistribution of lead from different body compartments and the slow release from bone, a process governed by bone turnover as described in studies of skeletal lead kinetics in which bone stores were shown to sustain circulating lead concentrations for years after exposure had ceased (25, 27, 29, 30). The relationship between chelation and the skeletal burden warrants nuance. The WHO similarly emphasizes that chelation therapy primarily removes lead from blood and soft tissues, whereas significant skeletal stores may result in subsequent remobilization and recurrent increases in blood lead concentrations after treatment (5). Taken together, these observations suggest that chelation attenuates but does not abolish the skeletal reservoir. This reflects the compartmentalized distribution of lead in the body and highlights the challenge of achieving sustained reduction of total body burden in chronically exposed children.

After one year of chelation therapy in a symptom-free patient, we discontinued treatment once the serum lead level reached 304.1 ug/L (Figure 2). The patient remained free of clinical symptoms. Continued long-term monitoring remains essential, however, as developmental, neurocognitive, and behavioral sequelae of childhood lead exposure may become apparent only later in life. Longitudinal studies have demonstrated persistent cognitive and neuropsychological impairments decades after childhood lead exposure, including deficits in executive function, memory, verbal abilities, and fine motor performance (31–33). Accordingly, the absence of clinical symptoms at the end of chelation therapy does not necessarily indicate complete recovery from the biological or developmental consequences of lead exposure. Long-term surveillance is particularly relevant after substantial exposure, although specific follow-up recommendations for children with very high blood lead concentrations remain limited. The WHO recommends repeated blood lead measurements after cessation of treatment, with the frequency depending on the initial blood lead concentration, severity of poisoning, and potential ongoing exposure (5). Clinical experience with other persistent sources of lead exposure, such as retained lead-containing metallic fragments, further illustrates the importance of prolonged monitoring: Patients with retained lead-containing fragments may develop systemic lead toxicity years after the initial exposure, and repeated blood lead measurements have been recommended in selected high-risk cases (34–37). Although the underlying exposure scenario differs from that in the present case, both involve a persistent internal lead depot capable of sustain lead release into the circulation. These observations further support monitoring the long-term decline in blood lead concentrations rather than relying on a single reassuring measurement after removal of the external source.

This case highlights both the importance of meticulous environmental source identification and the prolonged biological consequences of chronic lead exposure, even after successful elimination of the external source.

## Conclusion

In summary, lead poisoning should still be considered as a differential diagnosis, even though its incidence has been declining for years across Europe. The diagnosis can be challenging, as lead poisoning may mimic the symptoms of porphyria and lead-induced encephalopathy can resemble PRES on cranial MRI scan. Under chelation therapy, the patient has become symptom-free for 12 months and is developing appropriately for his age. However, potential long-term consequences, such as growth impairment, reduced intelligence or fertility disorders, cannot be estimated at present, especially given that lead stored in bone may have a half-life of more than 20 years.

## Data Availability

The datasets presented in this article are not readily available because of ethical and privacy restrictions. Requests to access the datasets should be directed to the corresponding author.
