# Supplementary material for: Heavy metal – habitual licking of a lead tape as rare cause of chronic lead poisoning: a case report
Source: Front Pediatr. 2026 Sep 9;14:1909793. doi: 10.3389/fped.2026.1909793 (PMC13597698; doi:10.3389/fped.2026.1909793)
Supplement: Supplementary file 1 [file Datasheet1.pdf]

Suppl. Table 1: Laboratory values upon admission.

Elevated/reduced parameters are marked in bold.

	patient's value	reference value
Leukocytes	6.8/nl	4.5-13-5/nl
Erythrocytes	**3.7/pl**	3.8-5.4/pl
Hemoglobin	**9.1 g/dl**	10.7-16.5 g/dl
Hematocrit	**26.9 %**	36-51%
MCV	**73.7 fl**	80-96 fl
MCH	**24.9 pg**	27-33 pg
Thrombocytes	**387/nl**	200-360/nl
Quick	70 %	<10 nmol/24h
Bilirubin (total)	**1.44 mg/dl**	< 1.0 mg/dl
Bilirubin (direct)	**0.67 mg/dl**	< 0.3 mg/dl
AST	**62 U/l**	19-48 U/l
ALT	**76 U/l**	7-44 U/l
Gamma GT	**62 U/l**	12-25 U/l
Urea	19 mg/dl	15-36 mg/dl
Creatinine	0.62 mg/dl	0.39-0.73 mg/dl
CRP	<0.6 mg/l	< 5.0 mg/l
Ferritin	**431 ng/mL**	25-112 ng/ml
Transferrin saturation	36.4 %	7-43 %
Soluble transferrin receptor	**5.59 mg/l**	2.2 – 5.0 mg/l

Suppl. Table 2: Lead concentration in lead chain and curtain.

**31.10.2024**		
**sample**	**lead chain**	**curtain**
concentration	mg/g	mg/g
lead	86.9	0.4
